# Dynamics of Antibiotic Resistance Gene Profiles in Captive Forest Musk Deer (*Moschus berezovskii*) Along a Breeding Duration Gradient

**DOI:** 10.3390/biology15171538

**Published:** 2026-09-04

**Authors:** Lijuan Suo, Kun Bian, Jie Tang, Feiran Li, Shuo Chang, Tianhao Yang, Xianmiao Zhao, Wuzi Dong, Kuo Sun, Chao Yang

**Affiliations:** 1 Shaanxi Key Laboratory of Qinling Ecological Security, Shaanxi Institute of Zoology, Xi’an 710032, China; 2 College of Animal Science and Technology, Northwest A&F University, No. 22 Xinong Road, Xianyang 712100, China; 3 Shaanxi Center for Animal Disease Prevention and Control, Xi’an 710018, China

**Keywords:** forest musk deer (*Moschus berezovskii*), antibiotic resistance genes, mobile genetic elements, co-localization, One Health

## Abstract

Background: To conserve wild populations and ensure a sustainable supply of musk, China initiated the captive breeding of forest musk deer. The temporal dynamics of gut antibiotic resistance gene (ARG) profiles in captive forest musk deer along a breeding duration gradient remain poorly characterized. Methods: In this study, we employed metagenomic sequencing to systematically characterize the profiles and potential mobility of ARGs. Samples were divided into short-term, medium-term and long-term groups according to breeding durations. Results: A total of 331 ARG subtypes and 71 mobile genetic element (MGE) subtypes were annotated across all samples. ARG Shannon diversity differed overall across groups (Kruskal-Wallis, *p* = 0.03); Bonferroni-adjusted Dunn’s test showed no significant pairwise differences. PCoA (Bray–Curtis) demonstrated distinct separation of the ST group (*p* = 0.002), and shared core ARG subtypes gradually increased with extended breeding years. A strong positive correlation between ARG and MGE abundances was identified (r = 0.85, *p* = 0.0001). In total, 63 contigs carrying co-localized ARG-MGE complexes were recovered. The ST group contained the highest proportion of such contigs. The ST group displayed tight physical ARG-MGE linkage within 1–3 kb genomic intervals. Conclusions: Our results reveal that breeding duration is associated with the gut ARG characteristics of captive forest musk deer. Short-term captivity has higher ARG-MGE co-localization, suggesting a higher possibility of mobilization.

## Simple Summary

Forest musk deer (*Moschus berezovskii*) are protected captive medicinal animals. The temporal dynamics of gut antibiotic resistance gene profiles in captive forest musk deer along a breeding duration gradient remain poorly characterized. We collected fecal samples from deer farms with short, medium and long breeding histories and analyzed all genetic material from animal gut microbes. Farms with newly raised deer tended to harbor relatively more antibiotic resistance genes. These genes were more likely to move due to mobile genetic elements. Farms operating for many years had fewer unique resistance genes and formed stable shared gene profiles. Antibiotic resistance genes were found linked to mobile genetic elements on all farms. Our work indicates that short-term captive herds exhibit elevated co-localization of ARGs and MGEs, suggesting a higher possibility of mobilization.

## 1. Introduction

Antimicrobial resistance (AMR) has emerged as one of the most urgent global public health challenges, primarily driven by the widespread misuse of antibiotics in clinical, agricultural, and livestock farming practices [1,2,3]. This selective pressure facilitates the widespread dissemination of antibiotic resistance genes (ARGs) across human, animal, and environmental compartments [4,5]. Under the One Health framework, this creates an interconnected resistome [6]. The dissemination of ARGs relies primarily on two genetic pathways: vertical gene transfer mediated by bacterial clonal expansion and cross-taxa horizontal gene transfer (HGT) [6,7]. The latter is the predominant mechanism in microbial communities, driven by mobile genetic elements (MGEs) including plasmids, transposons, integrons, and insertion sequences [8,9]. ARG-MGE co-localization facilitates the cross-host HGT of resistance genes [9,10]. Animal gut microbiomes, especially in livestock, are major reservoirs of MGE-associated ARGs [11,12].

The forest musk deer (*Moschus berezovskii*) is listed as a Class I protected animal in China [13]. The musk secreted by male musk deer is a precious raw material for traditional Chinese medicine and a premium perfume ingredient [14]. Due to overhunting for musk production, wild populations have undergone sharp declines over the past century [15]. To conserve wild populations and ensure a sustainable supply of musk, China initiated the captive breeding of forest musk deer in 1958. This practice has now been widely implemented nationwide, serving as a core strategy for the ex situ conservation of the species. However, intensive captive management has led to high susceptibility to digestive disorders (e.g., diarrhea) and pneumonia in these animals [16,17,18]. Owing to the extremely short domestication history of forest musk deer and the lack of species-specific standardized veterinary protocols, empirical antimicrobial therapy remains a common practice on many farms [19]. Although direct farm-level antimicrobial usage data were not available in this study, studies have reported the frequent application of β-lactams, fluoroquinolones, and aminoglycosides in captive musk deer populations [20,21]. As a special economic animal, captive forest musk deer thus constitute a non-negligible reservoir of ARGs, demanding urgent attention.

The *Escherichia coli* isolates derived from forest musk deer harbor clinically relevant ARGs (e.g., *blaTEM*, *tetA*, and *sul1*), alongside MGEs such as *intI1* and *intI2* [15]. Metagenomic analysis revealed that, compared with wild forest musk deer, captive individuals exposed to antibiotics harbor ARGs with significantly higher diversity and abundance [19]. Notably, identical MGE-associated ARGs were detected in both forest musk deer and farm workers, suggesting a potential risk of horizontal transfer at the animal–human interface [19]. Despite these advances, current research remains largely confined to static comparisons between wild and captive populations. However, captivity is not a uniform condition. These populations differ markedly in management intensity, population density, dietary composition, and antimicrobial exposure [19,22]. Each of these factors imposes distinct and sustained selective pressures on the animal ARGs [19,23,24]. The dynamic trajectories of ARGs and MGEs along a temporal gradient of captive breeding years have yet to be characterized. Elucidating this temporal dimension is critical, as it may reveal whether ARG profiles converge, diversify, or stabilize with prolonged farming duration.

In this study, fecal samples of forest musk deer with different captive breeding years were collected and subjected to metagenomic sequencing. The main objectives were as follows: (1) to comparatively elucidate the distribution patterns of ARGs and MGEs; (2) to evaluate the potential role of MGEs in mediating ARG mobilization in the forest musk deer. By systematically revealing the temporal dynamics of the ARGs under captive conditions, this research enriches the theoretical framework of resistome dynamics in wildlife. Ultimately, it provides a scientific basis for managing resistance pollution in the standardized farming of forest musk deer, thereby contributing to the control of zoonotic resistance concerns under the global One Health strategy.

## 2. Materials and Methods

### 2.1. Animals and Sample Collection

A total of 15 forest musk deer farms were selected. According to different breeding years, samples were divided into three groups: a short-term group (ST, <5 years), a medium-term group (MT, 5–10 years), and a long-term group (LT, >10 years). Each group comprised five independent biological replicates (i.e., five distinct farms per group).

Within each farm, fresh fecal samples were collected from ten healthy adult forest musk deer. Only individuals that had not received antibiotic treatment for at least three months were included. The ten individual samples were collected from a single farm and then pooled in equal weights to generate one composite sample per replicate. Pooled sampling was employed to obtain sufficient microbial DNA yield and reduce sequencing costs, as the primary objective was to compare resistome profiles at the farm level across breeding durations. All samples were immediately placed in liquid nitrogen and subsequently stored at −80 °C for later use.

### 2.2. DNA Extraction and Metagenomic Sequencing

Total DNA was extracted from fecal samples using the E.Z.N.A.^®^ Stool DNA Kit (Omega Bio-tek, Norcross, GA, USA) according to the manufacturer’s protocols. High-quality DNA samples (OD260/280 = 1.8~2.2, OD260/230 ≥ 2.0) were used to construct a sequencing library. Metagenomic shotgun sequencing libraries were constructed and sequenced at Shanghai Biozeron Biological Technology Co. Ltd., Shanghai, China. The libraries were finally sequenced using a next-generation sequencing platform (MGI-T7) (Shanghai BIOZERON Biotech. Co., Ltd., Shanghai, China) with PE150 mode according to the standard protocols.

### 2.3. Quality Control

Raw sequence reads underwent quality trimming using Fastp (v0.23.1--length_required 75 --cut_right --cut_right_window_size 4 --cut_right_mean_quality 15) to remove adaptor contaminants and low-quality reads [25,26]. The quality-trimmed reads were then mapped against the forest musk deer reference genome (NCBI) using the BWA mem algorithm (parameters: -M -k 32 -t 16, http://bio-bwa.sourceforge.net/bwa.shtml (accessed on 23 November 2025)) [27]. After removing host-genome contaminations and low-quality data, the reads were labeled as clean reads and used for further analysis. Detailed per-sample sequencing and host-removal statistics are provided in Supplementary Table S1.

### 2.4. Metagenomic Assembly, ARG and MGE Analysis

From the clean sequence reads, a set of contigs of each sample was generated using MegaHit (v1.1.1-2-g02102e1) with “--min-contig-len 500” parameters [28]. Detailed assembly statistics for each sample are provided in Supplementary Table S2. The open reading frames (ORFs) of the assembled contigs were predicted using METAProdigal (v2.6.3) [29], and all ORFs generated a set of unique genes after clustering using CD-HIT (parameters: -n 9 -c 0.95 -G 0 -M 0 -d 0 -aS 0.9 -r 1) [30]. The longest sequence of each cluster was considered the representative sequence of each gene in the unique gene set. For ARG annotation, the representative gene sequences were aligned against the SARG database [31] using DIAMOND BLASTp (v0.9.22.123) with an E-value cutoff of 1 × 10^−5^, minimum identity of 70% (amino acid identity), and minimum query coverage of 40% (alignment coverage). For MGE annotation, BLASTn (v2.9.0+) was used against the mMGE database [32] with the following parameters: E-value ≤ 1 × 10^−5^, identity ≥ 70% (nucleotide identity), and query coverage ≥ 40% (alignment coverage). To eliminate technical biases caused by variable sequencing depth and gene length, we utilized Transcripts Per Kilobase Million (TPM) values generated by Salmon (v1.1.0) to quantify gene abundance [33,34]. Subsequently, TPM-normalized ARG or MGE abundances were log_10_-transformed and used for comparative analysis of differential abundance.

We considered ARGs and MGEs to be co-localized if they occurred in the same contig. The physical distance between adjacent ARG and MGE sequences was quantified to estimate the possibility of mobilization.

### 2.5. Health Risk Assessment of ARGs

ARGs were annotated for health-risk ranks according to the SARG database (SARG V3.2). The ranking framework proposed by Tong Zhang classifies ARGs into four risk levels based on anthropogenic enrichment, mobility potential, and host pathogenicity [35]. Rank I represents the current threat, Rank II represents the potential threat, and Ranks III and IV indicate relatively lower risks. Risk ranks were assigned by direct matching to predefined SARG categories and were based solely on ARG identity. No normalization was performed at the ranking step, as risk ranks are fixed categorical attributes of the database entries.

### 2.6. Statistical Analysis and Visualization

Alpha diversity (Shannon) was calculated using the R picante package (V1.8, https://github.com/skembel/picante (accessed on 22 March 2019)). Beta diversity was evaluated using Bray–Curtis distance matrices with 999 permutations, and PCoA was performed with the vegan package (v2.6-4). For statistical comparisons, normally distributed data were analyzed using one-way analysis of variance (ANOVA) followed by Tukey’s honest significant difference (HSD) post hoc test; non-normally distributed data were analyzed using the Kruskal–Wallis test followed by Dunn’s post hoc test with Bonferroni adjustment. For correlation analyses, Spearman’s rank correlation coefficient was used since the data did not meet normality assumptions; *p*-values were adjusted using the Benjamini–Hochberg procedure to control the false discovery rate. Graphs were generated using Origin 2021, GraphPad Prism 9.0, and the CFViSA platform (http://cloud.biomicroclass.com/CloudPlatform (accessed on 8 April 2026)).

## 3. Results

### 3.1. Profiling of ARGs

**Figure 1:**
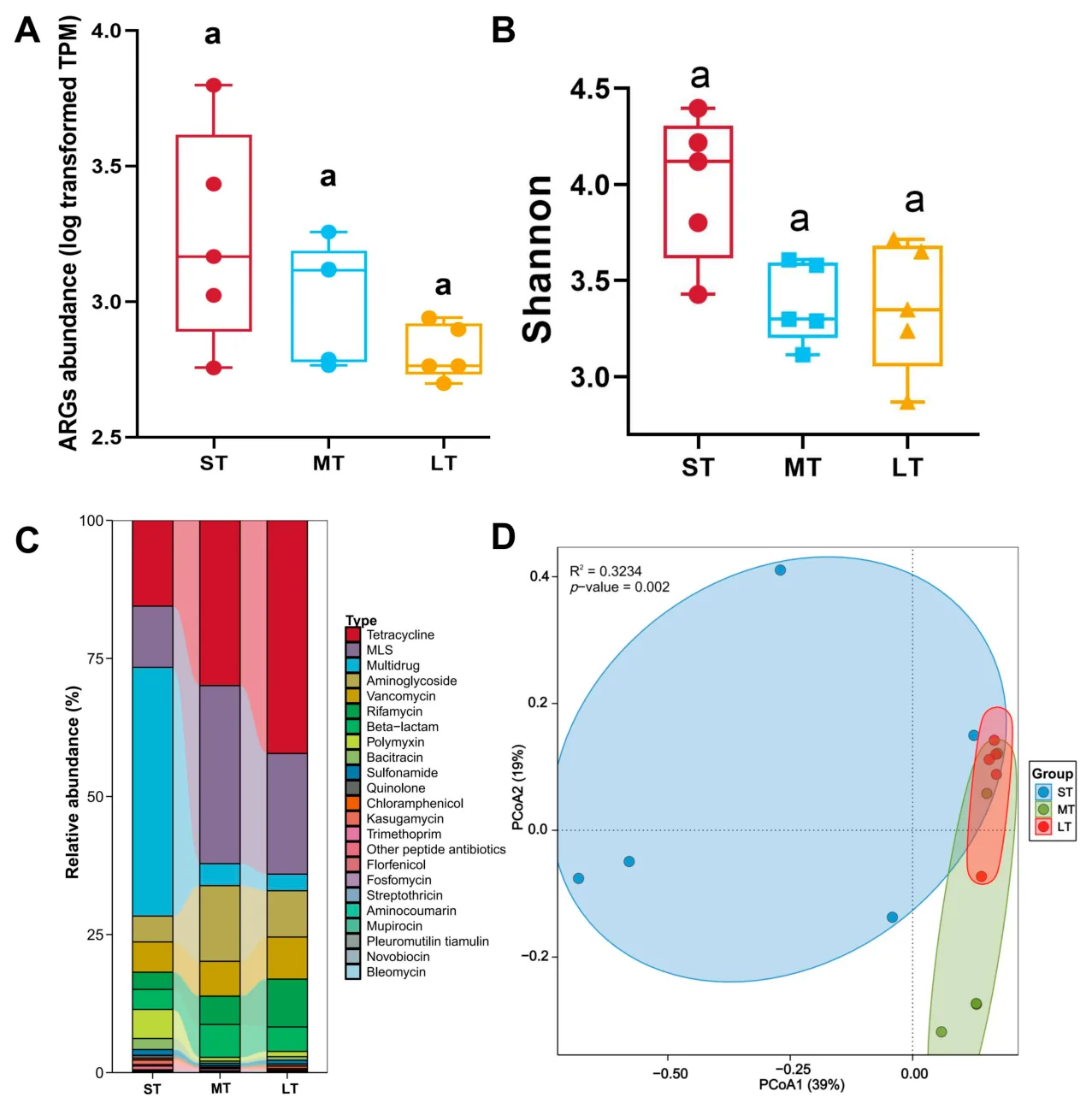
Composition and abundance of ARGs across different groups. (**A**) Comparison of ARG total abundance. (**B**) Comparison of Shannon diversity indices of ARGs. (**C**) Stacked bar chart of the relative abundances of ARG types. (**D**) PCoA plot of the composition of MGEs based on Bray–Curtis distances. Different lowercase letters denote significant differences between groups (*p* < 0.05), and identical letters indicate no significant difference.

A total of 23 ARG types and 331 subtypes were identified across the 15 samples. The ST group exhibited the highest total abundance of ARGs, while the LT group showed the lowest abundance of ARGs in the feces of forest musk deer; nevertheless, no significant differences in total abundance were observed among the three groups (*p =* 0.10) (Figure 1A). The predominant resistance types included tetracyclines, multidrug resistance, MLS, aminoglycosides, vancomycin, β-lactams, polymyxins, bacitracin, sulfonamides, and quinolones (Figure 1C). Among these, tetracyclines, MLS, and multidrug resistance genes were the most prevalent, representing 71.6%, 66.1%, and 67.1% of the total ARGs in the ST, MT, and LT groups, respectively.

To further elucidate the distribution patterns, we conducted α- and β-diversity analyses across the three groups. The Kruskal–Wallis test revealed an overall difference in ARG Shannon diversity among the three groups (*p* = 0.03), yet Dunn’s post-hoc test with Bonferroni adjustment detected no significant pairwise differences, despite a tendency for higher values in the ST group. Additionally, PCoA based on Bray–Curtis distances revealed a clear separation between the ST group and the other two groups (*p* = 0.002) (Figure 1D).

### 3.2. Shared Characteristics of ARGs and Identification of High-Risk ARGs

**Figure 2:**
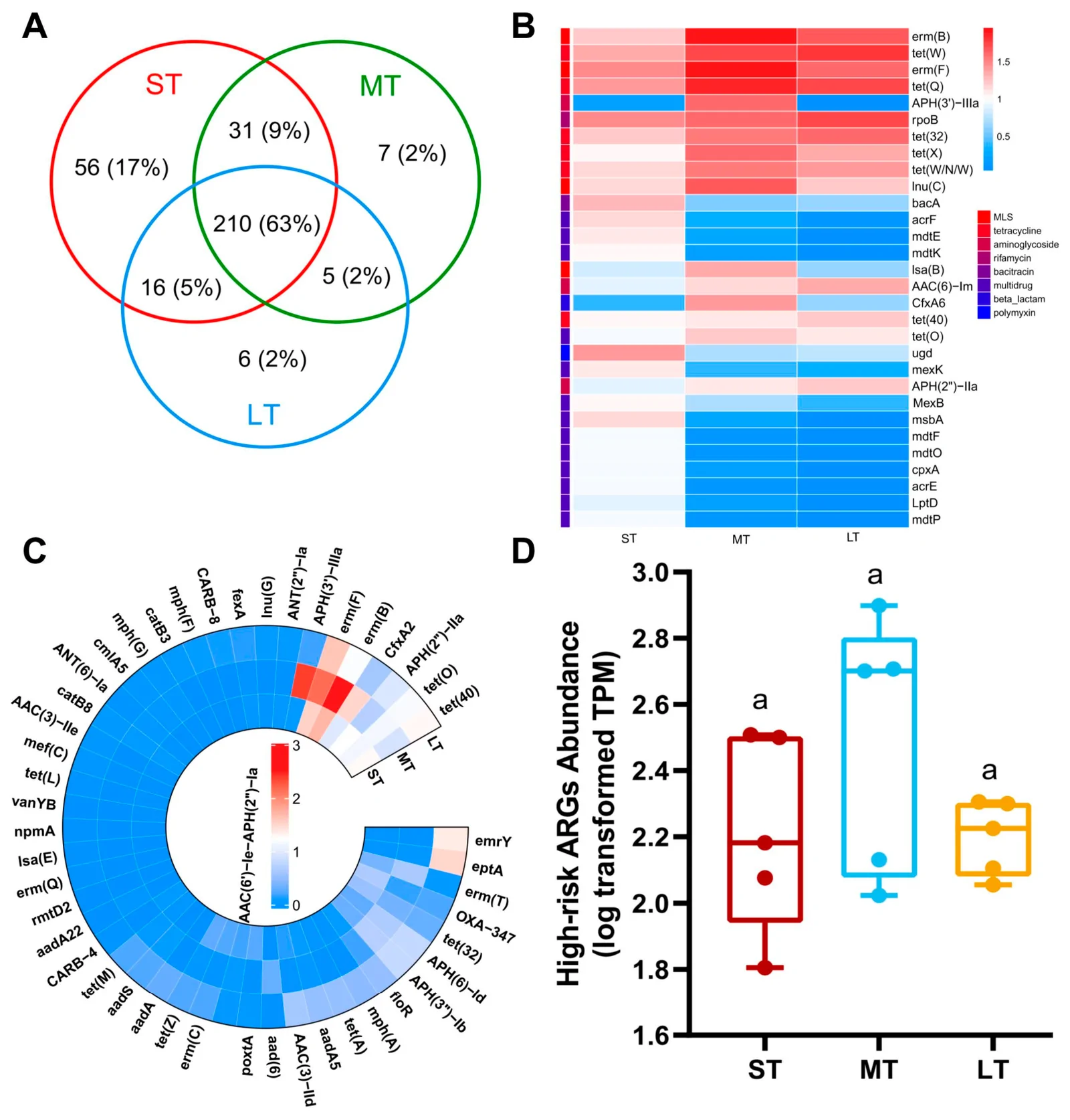
Shared characteristics and subtypes of ARGs across different groups. (**A**) Venn diagram of unique and shared ARGs. (**B**) Heatmap of the abundance of dominant ARGs (log-transformed TPM). (**C**) A circular heatmap for the abundance (log-transformed) of high-risk ARGs. (**D**) Comparison of high-risk ARG abundance. Different lowercase letters denote significant differences between groups (*p* < 0.05), and identical letters indicate no significant difference.

Shared subtypes made up the majority of ARGs across all groups. Their relative abundance increased from 67.1% in the ST group to 83.0% and 88.6% in the MT and LT groups, respectively. Conversely, despite the ST group hosting 56 unique subtypes, the MT and LT groups showed minimal specialization, with only 7 and 6 specific subtypes (Figure 2A). The heatmap (Figure 2B) depicts the relative abundance profiles of the top 30 ARG subtypes. Among these, five subtypes, *lnu*(*C*) (*p* = 0.01), *rpoB* (*p* = 0.01), *CfxA6* (*p* = 0.02), *APH*(*3′*)-*IIIa* (*p* = 0.02), and *ugd* (*p* = 0.03), showed significant differential abundance across the ST, MT, and LT groups.

ARG risk ranking analysis was performed to assess the risks of ARGs and identify ARGs with substantial threats to public health. From a clinical risk perspective, a total of 31 Rank I ARG subtypes and 16 Rank II ARG subtypes were detected (Figure 2C). These ARGs were classified into nine antibiotic resistance gene categories: aminoglycosides, β-lactams, chloramphenicols, florfenicol, MLS, multidrug resistance, polymyxins, tetracyclines, and vancomycins (Supplementary Table S3). The *erm*(*B*), *erm*(*F*), and *APH*(*3′*)-*IIIa* genes were the dominant high-risk ARG subtypes across all groups. The MT group exhibited the highest relative abundance of high-risk ARG subtypes, followed by the ST and LT groups; however, no statistically significant differences were detected between these groups (*p* = 0.50) (Figure 2D).

### 3.3. Abundance and Composition of MGEs

To characterize the mobilome, which potentially facilitates mobilization, we analyzed the composition and distribution of MGEs across the three groups. A total of 71 MGE subtypes were identified, categorized into five major types: transposase, integrase, plasmid, IS element, and ist.

**Figure 3:**
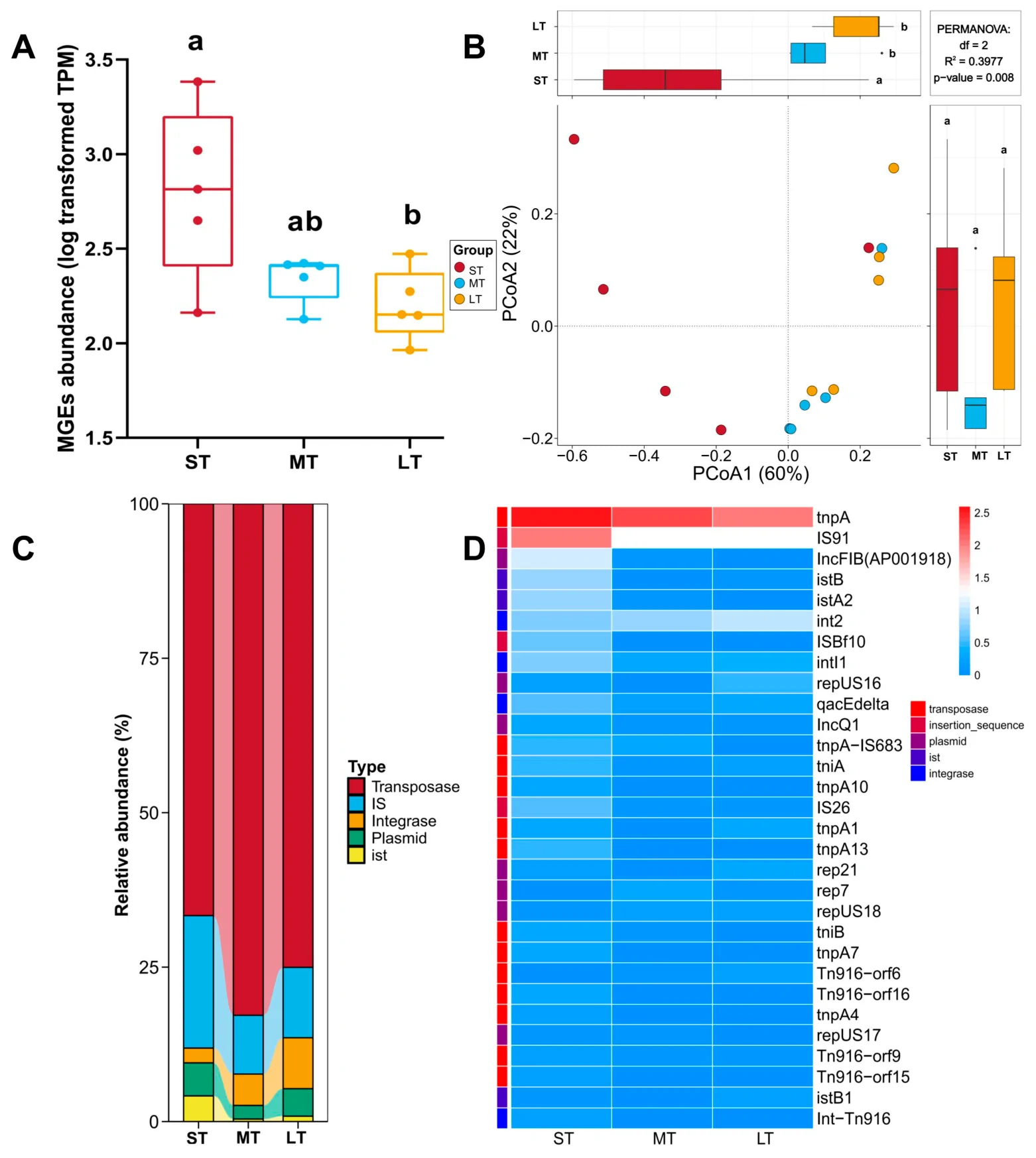
Composition and distribution of MGEs across different groups. (**A**) Comparison of MGE total abundances. (**B**) PCoA plot of the composition of MGEs based on Bray–Curtis distances. (**C**) Stacked bar chart of the relative abundances of MGE types. (**D**) Heatmap of the abundance of dominant MGE subtypes (log-transformed TPM). Different lowercase letters denote significant differences between groups (*p* < 0.05), and identical letters indicate no significant difference.

The total abundance of MGEs differed significantly among groups (*p* = 0.04) (Figure 3A). Moreover, their community composition exhibited distinct patterns (*p* = 0.008) (Figure 3B). Transposases and IS elements dominated all groups. The ST group displayed a relatively high proportion of IS elements and low proportions of integrase compared to the MT and LT groups (Figure 3C). The relative abundance heatmap of the top MGE subtypes further confirmed these compositional shifts, with notable variations in *tnpA* (*p* = 0.04) and *IS26* (*p* = 0.03) abundances (Figure 3D).

### 3.4. Correlation Between ARGs and MGEs

**Figure 4:**
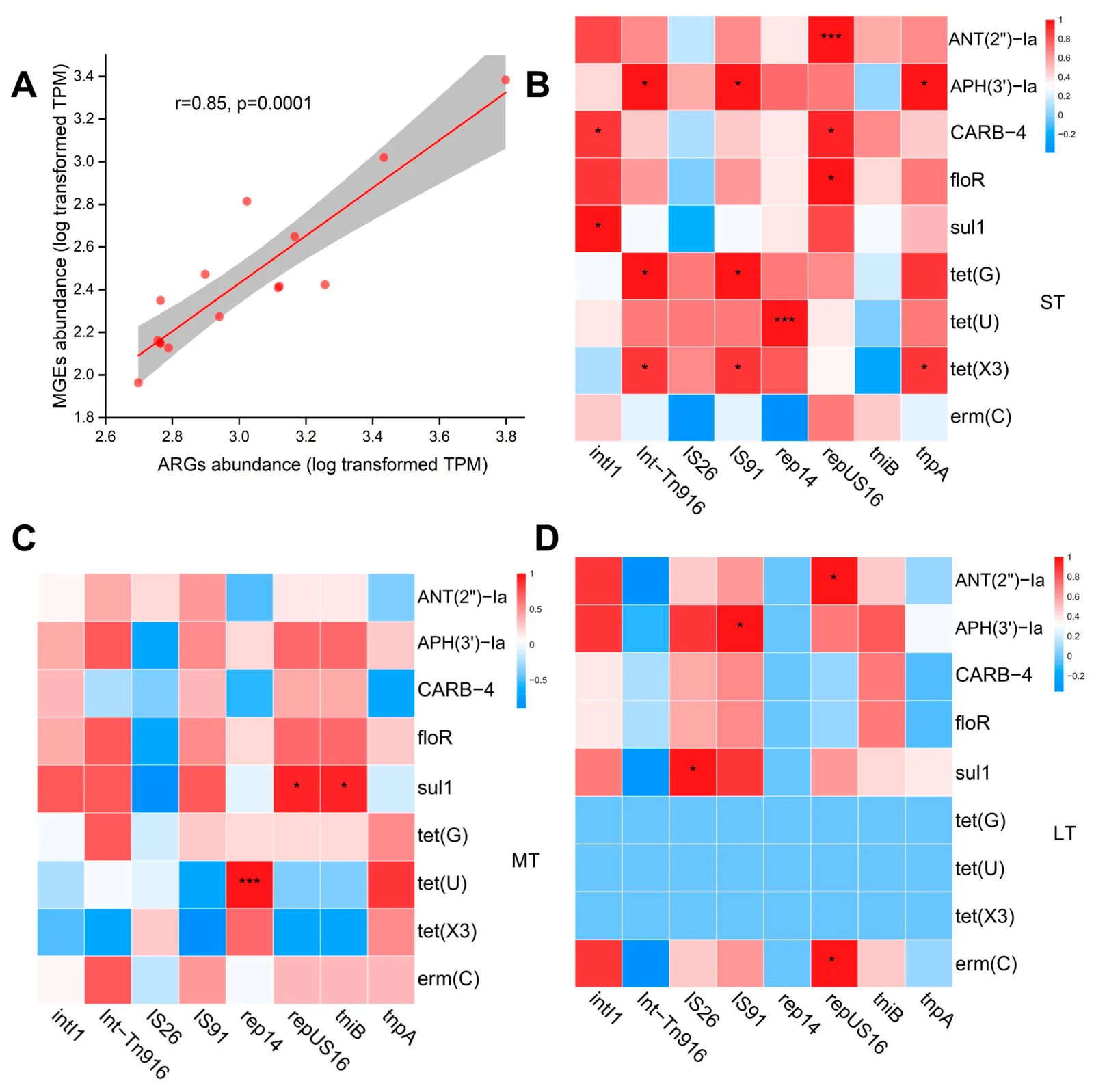
Correlation between ARGs and MGEs across different groups. The solid red line indicates the linear regression fit, and the gray shaded area denotes the 95% confidence interval for the regression line. (**A**) The correlation analysis between the total abundances of ARGs and MGEs. (**B**–**D**) The correlation analysis between the abundances of important ARG and MGE subtypes in the ST, MT and LT groups. Symbols denote significance levels: * *p* < 0.05, *** *p* < 0.001.

To evaluate the possibility of ARG mobilization in the fecal microbiota of forest musk deer, we analyzed the correlation between the total abundance of ARGs and the abundance of MGEs. Spearman’s rank correlation analysis revealed a strong positive correlation between total ARG and total MGE abundances (r = 0.85, 95% CI: 0.587 to 0.951, *p* = 0.0001) (Figure 4A), which is consistent with the potential for HGT but does not demonstrate active transfer; functional validation would be required to confirm actual HGT.

Subtype-level correlation analysis revealed distinct positive association patterns across the three groups. In the ST group, *ANT*(*2″*)-*Ia* was correlated with *repUS16* (r = 1.0, *p* = 0). Furthermore, *APH*(*3′*)-*Ia* and *tet*(*X3*) both displayed strong positive correlations with *Int-Tn916*, *IS91*, and *tnpA* (Figure 4B). In the MT group, *sul1* showed positive correlations with *repUS16* (r = 0.89, *p* = 0.04) and *tniB* (r = 0.89, *p* = 0.04), and *tet*(*U*) displayed a strong positive correlation with *rep14* (r = 1.0, *p* = 0) (Figure 4C). In the LT group, both *ANT*(*2″*)-*Ia* and *erm*(*C*) exhibited a strong positive correlation with *repUS16* (r = 0.97, *p* = 0.01) (Figure 4D).

### 3.5. Co-Occurrence Analysis of ARGs and MGEs

**Figure 5:**
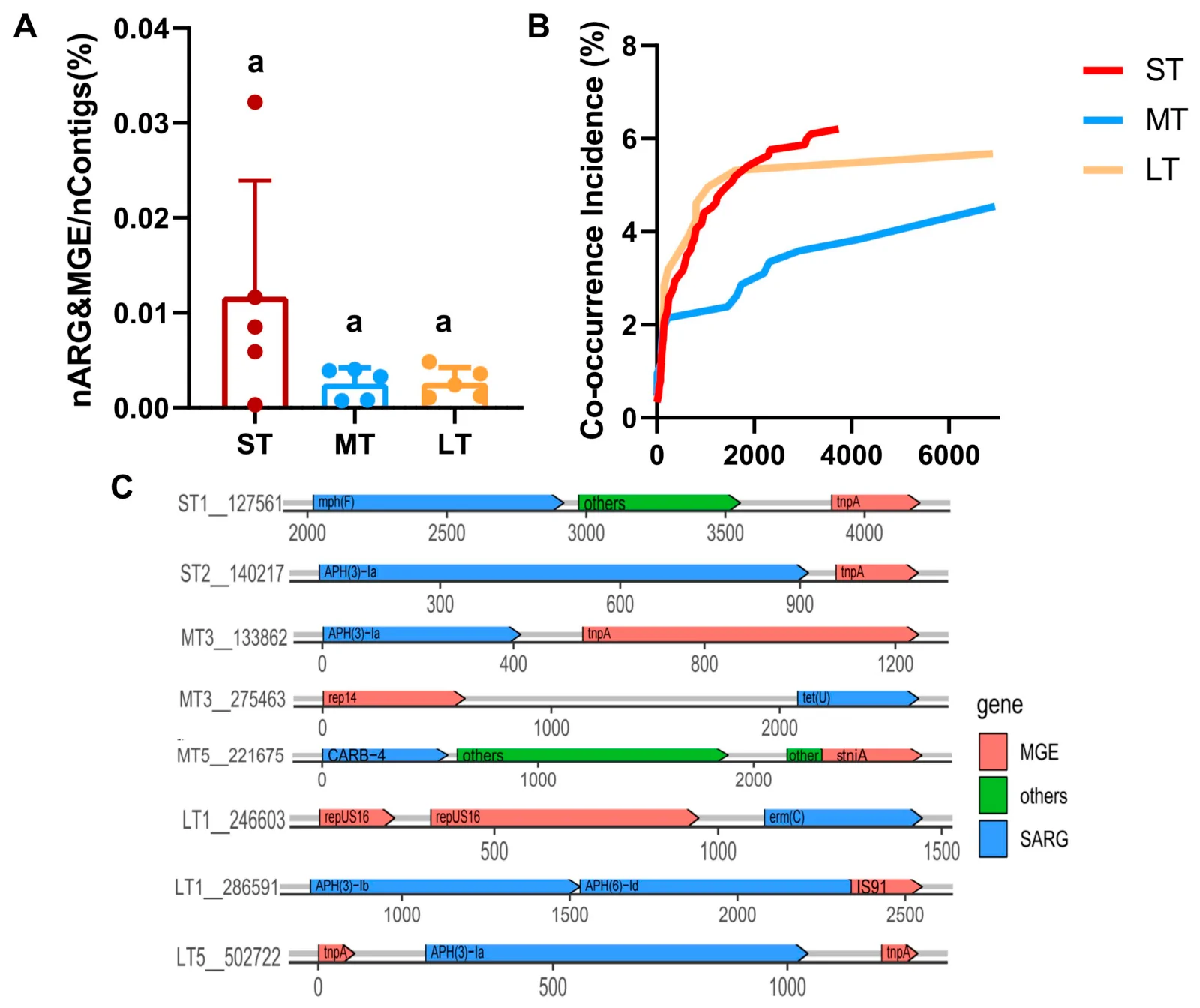
Co-occurrence of ARG and MGE combinations. (**A**) Comparison of the proportion of ARG-MGE overlapping clusters across different groups. (**B**) The co-occurrence incidence of ARGs and MGEs. (**C**) Arrangement patterns of ARG and MGE gene cassettes in different overlapping clusters. Each row represents one overlapping cluster. Different lowercase letters denote significant differences between groups (*p* < 0.05), and identical letters indicate no significant difference.

To further evaluate the potential mobility of ARGs across experimental groups, we characterized the co-localization patterns of ARGs and MGEs within assembled accessory contigs (ACCs). In total, 63 ARG-MGE co-localized contigs were identified, covering 49 ARG subtypes and 31 MGE subtypes (Supplementary Table S4). Among these annotated genes, 16 high-risk ARG subtypes were screened out, including *AAC*(*3*)-*IId*, *AAC*(*3*)-*IIe*, *aadA5*, *ANT*(*2″*)-*Ia*, *APH*(*3″*)-*Ib*, *APH*(*6*)-*Id*, *CARB*-*4*, *CfxA2*, *erm*(*C*), *erm*(*F*), *fexA*, *floR*, *mph*(*A*), *mph*(*F*), *tet*(*A*), and *tet*(*M*). ARG-MGE co-localized contigs were ubiquitously detected in the LT, MT and ST groups, but there was no significant difference (*p* = 0.19); notably, the ST group harbored the highest relative proportion of ACCs carrying paired ARGs and MGEs (Figure 5A). However, the cumulative co-occurrence frequency of ARGs and MGEs increased with increasing physical distance across all groups (Figure 5B). While the LT group exhibited a slightly higher incidence at the shortest distance (0.48% at 6 bp), the ST group showed the steepest accumulation at intermediate distances (~1000–3000 bp), ultimately reaching the highest final frequency (6.21% at 3732 bp). In contrast, the MT group displayed a more gradual increase, peaking at 5.67% (6892 bp), whereas the LT group maintained the lowest overall profile, with a maximum of 4.55% (6940 bp). These results suggest higher ARG-MGE co-localization in the ST group, indicating higher potential mobility.

To provide direct genomic evidence, we extracted representative contigs in which ARGs and MGEs were physically linked (Figure 5C). Physical linkage analysis revealed tight genomic proximity (typically <1 kb) between ARG-MGE pairs such as *APH*(*3′*)-*Ia*-*tnpA*, *rep14*-*tet*(*U*), and *repUS16*-*erm*(*C*), which is fully consistent with the significant positive correlations observed in the heatmaps (Figure 4B–D). *APH*(*3′*)-*Ia* was found co-localized with *tnpA* across all groups. In addition, high-risk genes such as *mph*(*F*), *CARB*-*4*, *APH*(*3″*)-*Ib* and *APH*(*6*)-*Id* are tightly physically linked to MGEs such as *tnpA* and *IS91*.

## 4. Discussion

The forest musk deer (*Moschus berezovskii*) is important for both biodiversity conservation and the traditional medicine industry [36,37]. In this study, we systematically characterized ARGs within the gut microbiome of captive forest musk deer. We identified 23 types of ARG with 331 subtypes from the samples. This result verifies that the gut of captive forest musk deer serves as an important reservoir of ARGs. The present study extends previous relevant research. Previous research revealed that captive forest musk deer exposed to antibiotics harbored a far more diverse resistome than their wild conspecifics [19]. Tetracyclines, MLS, and multidrug resistance genes were the predominant categories in this study. This profile aligns with the ARG characteristics observed in intensive farming systems and captive wildlife [38,39]. ARGs can remain in the environment long after antibiotic use stops, carried by mobile genetic elements [40,41]. Thus, the ARGs in our study are likely the result of farm-level factors, such as antimicrobial usage history, diet, and management practices, not just a quick response to recent drug use.

A key finding of this study is that the abundance of ARGs in the gut of *Moschus berezovskii* was negatively associated with breeding duration, although this trend was not statistically significant. This result is intriguing. Prolonged antibiotic exposure is generally expected to drive the accumulation of ARGs [42,43,44]. However, given the lack of detailed antibiotic usage records for each farm, we cannot attribute this decline solely to breeding duration; unmeasured farm-specific factors may also contribute. Pan et al. [45] reported a similar trend in fermentation-bed pig farming, finding significantly higher abundances of ARGs and MGEs in short-term fermentation beds than in long-term ones. The study also revealed that ARG diversity changes as the breeding time increases. The ST group had a relatively higher Shannon diversity index, and its β-diversity was clearly different from that of the MT and LT groups. This suggests that the elevated diversity in the ST group is associated with multi-source inputs, potentially encompassing inputs from environmental reservoirs (e.g., soil), consistent with early captivity colonization dynamics in wildlife [19,46,47]. Nevertheless, the pooled sampling design may not fully capture the extent of inter-individual variation in ARG diversity within each farm [48]. Therefore, the observed diversity differences should be interpreted at the farm-population level. The MT and LT groups exhibited convergent ARG profiles. Specifically, the Shannon index decreased, while the proportion of shared ARG subtypes rose markedly. This pattern matches what has been reported in captive wildlife under continuous antibiotic pressure, characterized by the convergence of α-diversity and the long-term persistence of a few core subtypes [49,50] (e.g., tetracycline, MLS, and multidrug resistance genes).

ARG risk depends not only on their abundance but, more importantly, on their mobility [51,52,53]. Our analysis revealed a significant positive correlation between ARGs and MGEs, suggesting an association rather than causation. In this study, the physical co-localization of ARGs (e.g., *mph*(*F*), *CARB*-*4*, *APH*(*3′*)-*Ib*, *APH*(*6*)-*Id*) and MGEs (e.g., *tnpA*, *IS91*) in accessory contigs provides genomic clues regarding their potential mobility [9,10]. It is important to note that co-localization suggests the possibility of mobilization but does not constitute direct evidence of active HGT. Functional validation via conjugation assays would be required to confirm actual HGT. However, these co-localizations were identified from pooled samples. Their detection only shows that the elements are present at the farm level, without revealing the proportion of individuals carrying these elements. This limits our ability to assess HGT at the population level. We cannot distinguish whether a single individual is carrying the elements or if they are widely disseminated within the farm.

Notably, although the ARGs in the LT group had converged to a stable state, the ST group showed the highest potential mobility. This is supported by the steepest increase in ARG-MGE co-occurrence within the 1000–3000 bp region and the highest proportion of contigs with co-localized genes. This suggests that long-term captivity may stabilize the core ARGs. However, beyond breeding duration, other farm-level factors, such as antimicrobial usage history, diet, and management practices, may contribute to this pattern, and their relative contributions cannot be fully disentangled from the current dataset.

In this study, we identified 16 high-risk ARGs in ARG-MGE co-localized contigs. Among them, *aph*(*3″*)-*Ib*, *aph*(*6*)-*Id*, *mph*(*A*), and *tet*(*A*) are especially noteworthy because they provide resistance to aminoglycosides, MLS, and tetracyclines. These are antibiotic classes heavily used in both human and veterinary medicine. Furthermore, previous studies have shown that these same ARGs are often shared among livestock, environmental, and clinical strains via *Tn3*, *IS26*, and *Inc*-*type* plasmids [9,54]. The presence of these high-risk ARGs in farm settings highlights the need for One Health surveillance [55]. The co-localization of these genes with MGEs implies their potential for co-mobilization. Together with prior evidence of shared ARG-MGE units between forest musk deer and their breeders [19], these findings suggest that forest musk deer farms may represent settings in which the potential for ARG exchange at the human–animal interface warrants attention; however, such an exchange was not demonstrated in the present study.

## 5. Conclusions

In summary, the present study indicates that breeding duration is associated with distinct ARG profiles in *Moschus berezovskii*. Long-term captivity was associated with a more stable, convergent resistome. Short-term captivity showed higher diversity and greater ARG-MGE co-localization, suggesting higher potential mobility. However, given the observational nature of this study and the lack of specific antimicrobial exposure data, these associations should be interpreted with caution. The physical linkage between high-risk ARGs and MGEs across all breeding groups underscores the urgent need for regular surveillance. Future studies integrating antibiotic usage data, farm management surveys, and functional assays are needed to establish causal relationships and validate actual HGT frequencies.

### Limitations of the Study

Several limitations should be acknowledged. First, the composite sampling strategy (pooling feces from ten individuals per farm) was adopted to balance sequencing costs and DNA yield, but it precludes quantification of within-farm inter-individual variation. Consequently, farm-level ARG diversity reflects a cross-host union rather than within-host diversity. Additionally, ARG-MGE co-occurrences in pooled samples may arise from different bacterial hosts, limiting HGT inference. Second, detailed records of antibiotic usage history, treatment frequency, and farm-specific management practices were not available. Breeding duration was therefore used as a proxy for cumulative anthropogenic pressure; however, we cannot exclude the possibility that these unmeasured farm-specific factors confounded the observed group differences. Our findings should thus be interpreted as associations rather than causal relationships. Third, while our co-localization analyses provide genomic evidence for potential HGT, culture-based functional assays (e.g., conjugation experiments) are still required to verify actual transfer frequencies.

## Data Availability

The original data presented in the study are openly available in the National Center for Biotechnology Information (NCBI) Sequence Read Archive (SRA) database (Accession Number: PRJNA1499942).

## Supplementary Materials

The following supporting information can be downloaded at: https://www.mdpi.com/article/10.3390/biology15171538/s1, Table S1: Sequencing, host removal, and read-mapping statistics for each pooled sample; Table S2: Metagenomic assembly statistics for each pooled sample; Table S3: High-risk ARGs of Rank I and Rank II; Table S4: Contigs carrying ARGs and MGEs.

## Abbreviations

The following abbreviations are used in this manuscript:

ARGs	Antibiotic Resistance Genes
MGEs	Mobile Genetic Elements
HGT	Horizontal Gene Transfer
AMR	Antimicrobial Resistance
