# Supplementary material for: Dynamics of Antibiotic Resistance Gene Profiles in Captive Forest Musk Deer (Moschus berezovskii) Along a Breeding Duration Gradient
Source: Biology (Basel). 2026 Sep 4;15(17):1538. doi: 10.3390/biology15171538 (PMC13564975; doi:10.3390/biology15171538)
Supplement: Supplementary file 1 [file biology-15-01538-s001.zip › biology-4498947-supplementary.pdf]

Dynamics of Antibiotic Resistance Gene Profiles in Captive Forest Musk Deer *(Moschus berezovskii)* Along a Breeding Duration Gradient

Lijuan Suo[1], Kun Bian[1], Jie Tang [1]* , Feiran Li[1], Shuo Chang[2], Tianhao Yang[2], and Wuzi Dong[2]

1 Shaanxi Key Laboratory of Qinling Ecological Security, Shaanxi Institute of Zoology, Xi'an 710032, China

2 College of Animal Science and Technology, Northwest A&F University, No. 22 Xinong Road, Xianyang712100, China

* Correspondence: yaya184@xab.ac.cn; Tel.: +86-029-83217271; Fax: +86-029-83217248

Summary Information

Number of pages: 4

Tables: S1-S4

Table S1 Sequencing, host removal, and read-mapping statistics for each pooled sample.

Sample ID	Raw reads (M)	Clean reads (M)	Clean rate (%)	Clean data (Gb)	Host-mapped reads (%)	Mapping rate to gene set (%)
ST1	73.91	72.06	97.49%	10.79	83.71	73.53
ST2	73.90	71.95	97.36%	10.75	63.96	69.20
ST3	72.62	70.77	97.46%	10.58	68.18	67.41
ST4	78.69	73.33	93.19%	10.95	96.16	64.38
ST5	88.27	85.94	97.35%	12.85	63.4	75.75
MT1	69.11	67.17	97.19%	10.03	52.58	68.15
MT2	69.12	67.31	97.37%	10.06	60.97	64.62
MT3	78.42	76.39	97.40%	11.42	63.02	70.22
MT4	81.62	79.50	97.40%	11.89	63.03	70.26
MT5	71.94	69.95	97.23%	10.45	57.71	59.21
LT1	66.70	64.94	97.36%	9.70	57.91	59.46
LT2	68.57	66.75	97.36%	9.96	48.66	63.03
LT3	68.53	66.71	97.35%	9.94	48.28	72.33
LT4	66.67	64.87	97.29%	9.69	49.19	64.19
LT5	72.57	70.75	97.50%	10.58	85.71	58.25

Table S2 Metagenomic assembly statistics for each pooled sample.

Sample ID	No. of contigs	Total length (bp)	N50 (bp)	Max contig (bp)
ST1	146232	204092435	1778	406538
ST2	268716	367235461	1654	557885
ST3	334571	417278596	1432	209347
ST4	418773	611253714	1918	595835
ST5	270833	470421942	2842	457538

MT1	283024	410850693	1857	324747
MT2	394573	502792804	1472	386251
MT3	390137	532286959	1637	386980
MT4	400094	550613367	1669	388873
MT5	383420	419125643	1094	432704
LT1	351153	375260720	1061	432716
LT2	331875	374244405	1189	319760
LT3	232203	331934999	1818	406813
LT4	289727	338617311	1252	425447
LT5	504406	590170026	1246	575520

Table S3 High-Risk ARGs of Rank I and Rank II.

Genes	Class	Risk Levels
AAC(3)-IId	aminoglycoside	Rank I ARG families
AAC(3)-IIe	aminoglycoside	Rank I ARG families
AAC(6)-Ie-APH(2)-Ia	aminoglycoside	Rank I ARG families
aad(6)	aminoglycoside	Rank I ARG families
aadA22	aminoglycoside	Rank I ARG families
aadA5	aminoglycoside	Rank I ARG families
ANT(6)-Ia	aminoglycoside	Rank I ARG families
APH(2")-IIa	aminoglycoside	Rank I ARG families
APH(3")-Ib	aminoglycoside	Rank I ARG families
APH(3')-IIIa	aminoglycoside	Rank I ARG families
APH(6)-Id	aminoglycoside	Rank I ARG families
CARB-4	beta_lactam	Rank I ARG families
CARB-8	beta_lactam	Rank I ARG families
catB3	chloramphenicol	Rank I ARG families
catB8	chloramphenicol	Rank I ARG families
cmlA5	chloramphenicol	Rank I ARG families
emrY	multidrug	Rank I ARG families
eptA	polymyxin	Rank I ARG families
erm(B)	MLS	Rank I ARG families
erm(T)	MLS	Rank I ARG families
floR	florfenicol	Rank I ARG families
lnu(G)	MLS	Rank I ARG families
lsa(E)	MLS	Rank I ARG families
mph(A)	MLS	Rank I ARG families
mph(F)	MLS	Rank I ARG families
poxtA	MLS	Rank I ARG families
rmtD2	aminoglycoside	Rank I ARG families
tet(A)	tetracycline	Rank I ARG families
tet(L)	tetracycline	Rank I ARG families
tet(M)	tetracycline	Rank I ARG families
vanYB	Vancomycin	Rank I ARG families

aadA	aminoglycoside	Rank II ARG families
aadS	aminoglycoside	Rank II ARG families
ANT(2")-Ia	aminoglycoside	Rank II ARG families
CfxA2	beta_lactam	Rank II ARG families
erm(C)	MLS	Rank II ARG families
erm(F)	MLS	Rank II ARG families
erm(Q)	MLS	Rank II ARG families
fexA	chloramphenicol	Rank II ARG families
mef(C)	MLS	Rank II ARG families
mph(G)	MLS	Rank II ARG families
npmA	aminoglycoside	Rank II ARG families
OXA-347	beta_lactam	Rank II ARG families
tet(32)	tetracycline	Rank II ARG families
tet(40)	tetracycline	Rank II ARG families
tet(O)	tetracycline	Rank II ARG families
tet(Z)	tetracycline	Rank II ARG families

Table S4 Contigs carrying ARGs and MGEs.

Sample	Contig	ARG	MGE
ST1	ST1__105249	*tet(G)*	*intI1;IS91;qacEdelta*
ST1	ST1__106045	*tet(X3)*	*IS91*
ST1	ST1__127561	*mph(F)*	*tnpA*
ST1	ST1__22774	*APH(3")-Ib;APH(6)-Id*	*tnpA*
ST1	ST1__33155	*cpxA*	*IS91*
ST1	ST1__3597	*MexD*	*tnpA*
ST1	ST1__3829	*dfrA1;SAT-2;ANT(3)-IIa*	*IS91;ISCR1*
ST1	ST1__54996	*tet(C)*	*tnpA*
ST1	ST1__56218	*qacH;ANT(2")-Ia*	*intI1*
ST1	ST1__69230	*APH(3)-Ia*	*ISCR1;tnpA*
ST1	ST1__79854	*sul1;floR*	*IS91;tnpA*
ST1	ST1__85224	*sul2*	*tnpA*
ST1	ST1__91753	*tet(M)*	*Tn916；Int-Tn916；Xis-Tn916*
ST1	ST1__99596	*adeR*	*ISCR1；IS26*
ST2	ST2__140217	*APH(3')-Ia*	*tnpA*
ST2	ST2__163761	*tet(G)*	*qacEdelta*
ST2	ST2__189044	*AAC(3)-IId*	*tnpA10*
ST2	ST2__189044	*TEM-1*	*tnpAcp2;tnpA*
ST2	ST2__195884	*sul2*	*tnpA*
ST2	ST2__202598	*cpxA*	*IS91*
ST2	ST2__20864	*sul1*	*tniB*
ST2	ST2__227470	*tet(A)*	*tnpA*
ST2	ST2__234686	*mph(F)*	*tnpA*
ST2	ST2__7805	*floR;sul1*	*IS91*
ST2	ST2__78663	*APH(6)-Id;APH(3")-Ib*	*IS91;tnpA*

ST3	ST3__123643	*sul1*	*tniB*
ST3	ST3__165909	*fexA*	*tnpA*
ST3	ST3__193972	*erm(C)*	*rep22*
ST3	ST3__23612	*sul2*	*IS91*
ST3	ST3__260529	*lnu(A)*	*rep21*
ST3	ST3__265764	*erm(C)*	*repUS16*
ST3	ST3__27531	*tet(33)*	*tnpA*
ST3	ST3__33566	*vanVB*	*Tn916*
ST3	ST3__62968	*APH(3")-Ib;APH(6)-Id*	*tnpA;IS91*
ST4	ST4__1086649	*tet(X);erm(F)*	*tnpA*
ST5	ST5__10153	*tet(B)*	*IS10*
ST5	ST5__131336	*mph(A);aadA5;sul1; dfrA17;qacEdelta1*	*tnpA*
ST5	ST5__166487	*sul2*	*tnpA*
ST5	ST5__219257	*mel*	*tnpA*
ST5	ST5__26924	*tet(A)*	*tnpA;tniA*
ST5	ST5__85503	*QnrS4*	*ISCR1;tnpA*
MT1	MT1__154477	*AAC(6)-Ib7*	*intI1*
MT1	MT1__189144	*qacEdelta1;sul1*	*tnpA*
MT1	MT1__81969	*mel*	*tnpA*
MT2	MT2__129726	*CfxA3*	*tnpA*
MT3	MT3__133862	*APH(3')-Ia*	*tnpA*
MT3	MT3__275463	*tet(U)*	*rep14*
MT3	MT3__64164	*sul2*	*tnpA*
MT3	MT3__64537	*CfxA2*	*tnpA*
MT4	MT4__283866	*CfxA2*	*tnpA*
MT5	MT5__221675	*CARB-4*	*tniA*
MT5	MT5__359571	*sul1*	*IS91*
LT1	LT1__197484	*sul1*	*intI1；tniB；tniA*
LT1	LT1__246603	*erm(C)*	*repUS16*
LT1	LT1__286591	*APH(3")-Ib;APH(6)-Id*	*IS91*
LT1	LT1__304414	*floR*	*IS91*
LT3	LT3__108133	*AAC(3)-IIe*	*tnpA1133；tnpA；IS91*
LT4	LT4__112317	*sul1*	*tnpA；intI1*
LT4	LT4__276363	*CfxA2*	*tnpA*
LT5	LT5__11468	*lnu(A)*	*rep21*
LT5	LT5__242072	*erm(C)*	*repUS16*
LT5	LT5__502722	*APH(3')-Ia*	*tnpA*
LT5	LT5__92623	*APH(6)-Id;APH(3")-Ib*	*tnpA*
